# Site fidelity of migratory shorebirds facing habitat deterioration: insights from satellite tracking and mark-resighting

**DOI:** 10.1186/s40462-023-00443-9

**Published:** 2023-12-21

**Authors:** Ying-Chi Chan, David Tsz Chung Chan, T. Lee Tibbitts, Chris J. Hassell, Theunis Piersma

**Affiliations:** 1 Department of Coastal Systems, NIOZ Royal Netherlands Institute for Sea Research, P.O. Box 59, 1790 AB Den Burg, Texel, The Netherlands; 2 Rudi Drent Chair in Global Flyway Ecology, Conservation Ecology Group, Groningen Institute for Evolutionary Life Sciences (GELIFES), University of Groningen, P.O. Box 11103, 9700 CC Groningen, The Netherlands; 3 Alaska Science Center, U. S. Geological Survey, 4210 University Drive, Anchorage, AK 99508 USA; 4 Global Flyway Network, PO Box 3089, Broome, WA 6725 Australia; 5 Australasian Wader Studies Group, PO Box 3089, Broome, WA 6725 Australia; 6 BirdEyes, Centre for Global Ecological Change at the Faculties of Science and Engineering and Campus Fryslân, University of Groningen, Zaailand 110, 8911 BN Leeuwarden, The Netherlands; 7 Center for East Asian–Australasian Flyway Studies, School of Ecology and Nature Conservation, Beijing Forestry University, Beijing, China

**Keywords:** Bird migration, Seasonality, Waders, Yellow Sea, East Asian–Australasian Flyway

## Abstract

**Background:**

Site fidelity, the tendency to return to a previously visited site, is commonly observed in migratory birds. This behaviour would be advantageous if birds returning to the same site, benefit from their previous knowledge about local resources. However, when habitat quality declines at a site over time, birds with lower site fidelity might benefit from a tendency to move to sites with better habitats. As a first step towards understanding the influence of site fidelity on how animals cope with habitat deterioration, here we describe site fidelity variation in two species of sympatric migratory shorebirds (Bar-tailed Godwits *Limosa lapponica* and Great Knots *Calidris tenuirostris*). Both species are being impacted by the rapid loss and deterioration of intertidal habitats in the Yellow Sea where they fuel up during their annual long-distance migrations.

**Methods:**

Using satellite tracking and mark-resighting data, we measured site fidelity in the non-breeding (austral summer) and migration periods, during which both species live and co-occur in Northwest Australia and the Yellow Sea, respectively.

**Results:**

Site fidelity was generally high in both species, with the majority of individuals using only one site during the non-breeding season and revisiting the same sites during migration. Nevertheless, Great Knots did exhibit lower site fidelity than Bar-tailed Godwits in both Northwest Australia and the Yellow Sea across data types.

**Conclusions:**

Great Knots encountered substantial habitat deterioration just before and during our study period but show the same rate of decline in population size and individual survival as the less habitat-impacted Bar-tailed Godwits. This suggests that the lower site fidelity of Great Knots might have helped them to cope with the habitat changes. Future studies on movement patterns and their consequences under different environmental conditions by individuals with different degrees of site fidelity could help broaden our understanding of how species might react to, and recover from, local habitat deterioration.

**Supplementary Information:**

The online version contains supplementary material available at 10.1186/s40462-023-00443-9.

## Background

The year-to-year return of migratory animals has long been a source of wonder for humans [1]. Methods to identify individuals, such as marking birds individually using metal rings [2], lead to the discovery that not only the same species, but often the same individual returned to the same place year after year [3–6]. Ecologists have used the terms site fidelity, faithfulness, or philopatry for this tendency to return to a previously visited site. A wide range of taxa show fidelity to their breeding sites (philopatry), and for migratory species, site fidelity to stopover and non-breeding sites is also common [7–12].

Site fidelity is advantageous in circumstances where animals can benefit from their previous knowledge on the distribution of food resources, safe resting locations and predation danger; over time, site-faithful individuals may attain dominance over the best and safest local sites and resources [3, 13–15]. Site-faithful behaviour is beneficial when the environment is stable and predictable but it can also confer advantages in variable environments and lead to a higher lifetime fitness if animals are long-lived enough to weather years of unfavourable outcomes [16, 17].

Habitat loss and deterioration are major threats to migratory populations worldwide [18]. Strong site fidelity can be maladaptive [19–21] in cases where animals do not move to other habitats even if local habitat quality decreases. Moreover, if the distribution of high- and low-quality habitats shifts between years, it would be maladaptive for animals to base their decisions to stay or switch habitats on past experience [22]. Low site fidelity strategies that lead to a high propensity to move to alternative habitats when the original habitat deteriorates can be adaptive. This would involve migratory animals making decisions of staying or switching based on current environmental conditions, and engaging in behaviours that lower the cost of moving, e.g. by collecting information on alternative habitats, which would reduce the search time for alternatives and the risks involved. Therefore, the degree of site fidelity may influence how animals are impacted by habitat deterioration. A first step to understanding this relationship is to describe site fidelity variation in populations that occur in places with deteriorating habitat conditions. For migratory animals that traverse places of different degrees of habitat deterioration in their annual cycle, currently little is known regarding how site fidelity variation persists across places and seasons.

Here we explore interspecific variation in site fidelity of two migratory shorebird species facing habitat deterioration, Bar-tailed Godwit *Limosa lapponica* and Great Knot *Calidris tenuirostris*. During their annual migration from Northwest Australia to breeding areas in the East Russian Arctic, both species rely on major staging sites in coastal wetlands of the Yellow Sea [23, 24, 30], a region with rapid habitat loss and deterioration [25–27]. Both species show declines in survival rates and numbers [28, 29]. To compare site fidelity between the two species, we focus on two key periods of their annual cycle when they co-occur at the same coastal wetlands: (1) during the non-breeding season (austral summer / boreal winter), when these species are at their final non-breeding destination in Northwest Australia and (2) during migration at their main staging area (i.e. used for the longest time), along the coast of the Yellow Sea [23, 24, 30].

Site fidelity of birds is usually inferred from recapturing or resighting marked individuals [31–34], but inference is often limited by the inability to assign a cause for unobserved birds. For example, unsighted birds could have moved to unsurveyed sites (true site infidelity), have died (mortality) or have gone unobserved due to detection issues [35]. These limitations can be overcome with remote tracking of bird movements with global coverage, e.g. an Argos satellite tag or GPS tag. As a step towards a multi-species comparison of site fidelity in different environments using all available data types, we investigate difference in site fidelity between Bar-tailed Godwits and Great Knots with two types of data, tracked itineraries of satellite-tagged individuals and resightings of marked individuals, and examine how data types could affect the patterns inferred.

## Methods

### Bird marking and resighting

Individual marking of the study species was conducted at Roebuck Bay (18.1°S, 122.3°E) and Eighty Mile Beach (19.4°S, 121.3°E), Northwest Australia, two major non-breeding sites in the East Asian–Australasian Flyway for these species [36], each year in 2006–2019, from February to March, and from June to December. The birds were captured with cannon nets, measured and marked with unique combinations of colour-bands and a flag on their tibia or tarsi [28]. Birds were aged by the Australian method into 1st year, 2nd year and 3rd year or older (adults) based on plumage characteristics and moult scores.

On the northern shores of Roebuck Bay, throughout the non-breeding period (August to mid-April), 2007–2020, observations of banded birds (i.e. resightings) were conducted by experienced observers using 20–60 × zoom telescopes, several times a week during the 4-h daytime high-tide period. Most observations (~ 90%) were conducted at high-tide roost sites at the northern beaches. The available shoreline for birds to roost is about 9 km long and consists of sandy beaches interspersed with small rocky areas and roost choice is affected by tide height, disturbance and microclimate [37]. About 10% of the time, observations were done during in-coming tides while birds were feeding on the mudflats and being pushed towards the shore by the tide bringing them close enough for observers on shore to record their colour-band combinations.

Dedicated resighting work was also conducted every April for three days, 2010–2017 on a 65 km section in the northern part of the 220 km long Eighty Mile Beach (mid-point = 19.4°S, 121.3°E, 190 km southwest of Roebuck Bay). In addition, incidental observations were obtained each year during population count surveys (6 days/yr. November and December 2006–2017 and 3 days/yr. 2018–2019) and bird catching expeditions (10–11 days/yr. in November/December 2007–2010 and February 2011–2020).

Numerous surveys of shorebirds were conducted during migration along the coasts of the Yellow Sea, during which observers reported sightings of marked Bar-tailed Godwits and Great Knots to banding organizations. Here we highlight the main surveys that targeted resighting banded individuals and from which we gleaned data. At Luannan Coast, Bohai Bay (39.1° N, 118.2° E), a key staging site of Great Knots [38], we conducted intensive resighting work of banded birds during the northward migration period (mid-April to early-June), 2010–2020 [39]. In the Yalu Jiang Estuary National Nature Reserve, Liaoning, China (39.8° N, 123.9° E), a key staging site of both species [40], Fudan University and Pūkorokoro Miranda Naturalists’ Trust conducted surveys during northward migration (mid-March to mid-May), 2010–2020. At 14 shorebird sites along the Chinese coast, surveys were carried out during northward migration (April to June), 2015–2017, with observers spending 2–3 field days at each site (see Additional file 1 for site coordinates). Additionally, incidental sightings were reported by birdwatchers or from shorebird surveys that did not focus on observing banded individuals (e.g. [41]). We compiled all these observations into our resighting history for each individually marked Great Knot and Bar-tailed Godwit for our analysis of site fidelity.

### Satellite transmitter deployments

In September and October 2014–2016, we deployed 4.5 g and 9.5 g solar Platform Terminal Transmitters (PTTs, Microwave Telemetry, USA) onto a subset of the Great Knots and Bar-tailed Godwits banded at Roebuck Bay to track their movements. PTTs were programmed to operate on a duty cycle of 8 h of transmission and 25 h off. Tags were attached to Bar-tailed Godwits with a Teflon leg-loop harness [42], and onto Great Knots using a body harness [43] made of elastic nylon (Elastan, Vaessen Creative, The Netherlands). Birds were released at their capture locations.

### Measures of site fidelity based on tracking data

For all tracking data collected, we kept all standard Argos locations (i.e. location classes 3, 2, and 1, 68th error percentiles < 2.5 km, [44]). For auxiliary locations (i.e. classes 0, A, B and Z, 68th error percentiles between 10 and 30 km), we removed implausible locations by applying the Hybrid Douglas filter [44]. The filtering parameters were set at 120 km/h for the maximum sustainable rate of movement and 10 km for the maximum redundant distance. We further accounted for spatial error in the Argos telemetry by fitting the tracking data with a continuous-time random walk state-space model with the ‘fit_ssm’ function in the ‘foiegras’ R package [45]. The state-space model incorporated the error ellipse information of the Kalman filter-based Argos locations, and the fitted locations from the model were used in the analysis of identifying migration stops and timing.

We measured fidelity to a ‘site’, which was defined as a cluster of habitats that an individual bird used daily for foraging and roosting [46]. Site fidelity within the non-breeding period was measured for 41 Great Knots and 24 Bar-tailed Godwits that were tracked from their release date (in September to November) to one week before the first departure date of a tracked conspecific (among all years, first departure date: 22 March for Great Knots, 4 April for Bar-tailed Godwits), so as to avoid including pre-migratory movements in this analysis. We calculated the proportion of birds that remained at a single site during this non-breeding period and described movements to any other sites. Site fidelity across non-breeding periods was also estimated for the 10 Great Knots and 9 Bar-tailed Godwits with complete migration tracks to and from the breeding grounds. For these individuals, we calculated the proportion of birds that returned to Roebuck Bay and described the movements of those that overwintered at other sites.

Seasonal site fidelity to migratory stopover sites in the Yellow Sea was measured from the first recorded migration of each tracked individual. As per design, the elastic nylon harness material on Great Knots degrades and breaks within a year of deployment which did not allow for calculation of between-year fidelity of Great Knots to migration sites. Therefore, to characterize seasonal site fidelity for a comparison between species, we determined whether birds re-visited the same northward migration site during the subsequent southward migration in their first tracked migration. We employed the following procedures to identify stopover sites per northward/southward migration per individual: (1) locations within the Yellow Sea with ground speed less than 5 km/h were identified as ‘stationary’, (2) the stationary locations were clustered into sites using R package ‘NbClust’, using the ‘Complete’ aggregation method and ‘silhouette’ index [47] to determine the optimal number of clusters, and (3) distance between the centroid of the cluster and each point assigned to the cluster was calculated. If > 5% of points are further than 25 km away from the centroid, clustering was performed again. The resulting sites contained points of which ≥ 95% were within 25 km of their centroid. If centroids of two sites were closer than 50 km, they were merged. We discarded sites that contained less than 3 stationary locations and those where the first and last recorded locations were less than 2 h apart. Departure times were extrapolated over the intervening travel distance between the last location at a stop and the next location, and arrival times were calculated in the same way. A site was defined as re-visited across seasons when a southward site’s centroid was within 50 km of a northward site’s centroid for a particular bird. The threshold distance of 50 km was chosen as it is large enough to cover the habitats that an individual bird moves through daily for foraging and roosting [46, 48] and matches the spatial resolution at which the band resightings were reported. This ensures that metrics calculated from the satellite tracking data are comparable to those from the resighting data.

In addition to reporting site fidelity as the proportion of birds that re-visited sites across seasons, we also present the degree of site fidelity at the individual level, measured as the proportion of time birds spent at re-visited sites relative to their total length of stay in the Yellow Sea during southward migration. We compare these proportions between the two species by fractional regression. To show the frequency of movements within sites in the Yellow Sea, we present the number of Yellow Sea sites used per individual during northward and southward migration. We compare this metric to the same one from the resighting data, to provide an understanding of the magnitude of any issues resulting from non-observed movements when analyzing resighting data.

### Measures of site fidelity based on resighting data

We measured site fidelity during the non-breeding period from resighting data of marked adults captured in June to December in Roebuck Bay. While individuals carrying a satellite transmitter were also marked, they were excluded as their movements are already part of the analysis described above. For site fidelity within the non-breeding period, we analyzed individuals with two or more sightings from 1 November until a week before the first departure of the tracked birds; this resulted in a sample of 641 Bar-tailed Godwits and 775 Great Knots from which we then calculated the number of sites where each individual was resighted. We grouped individuals with ≥ 2 sightings for multiple years into one datapoint to avoid pseudo-replication, taking the maximum number of sites it was observed within a single non-breeding period. Individuals observed in ≥ 2 sites reflected a move between sites within the non-breeding period. If an individual was observed at only a single site, it was considered to have stayed there for the entire non-breeding period, moved to an un-surveyed site, or moved to a surveyed site but not observed there. The nature of the data did not allow us to distinguish between these scenarios.

We then compared the proportions of individuals observed at one or two sites between the two species by a Fisher’s Exact test. The above analysis pertained to within-season movement between the resighting sites of Roebuck Bay and Eighty Mile Beach. However, a small proportion of individuals were also observed by researchers/birdwatchers at other sites in the flyway during the non-breeding season. We further examined the sighting history of these few individuals to understand these rare long-distance movements.

To measure site fidelity during the migration period in the Yellow Sea, we expanded our dataset to include birds marked at other sites in Australia because, to realistically assess site fidelity, our sample size of birds marked with unique colour-bands in Northwest Australia was too small (only 34 Bar-tailed Godwits and 135 Great Knots seen two or more times in the Yellow Sea within 2008–2017). Therefore, we included resighting data from birds marked (with a flag engraved with a unique letter-number code) by the Australian Wader Studies Group (AWSG) at four other sites throughout Australia, and the resighting data was collected by the field efforts described above and collated by the AWSG. The final dataset comprises resightings in the Yellow Sea from 2008 to 2017 of 173 Bar-tailed Godwits marked in Northwest Australia and Victoria, and 513 Great Knots marked in Northwest Australia, Queensland, Northern Territory and Victoria.

Movements between sites within the Yellow Sea within a migration season were described by the number of sites where an individual was resighted. We calculated this metric only for individuals that were resighted ≥ 2 times within a migration season and only for northward migration, because sample sizes were too low during southward migration (only 4 Bar-tailed Godwits and 6 Great Knots were resighted ≥ 2 times). We highlight how the imperfect nature of resighting data affects the results when we compare this metric with the satellite tracking data.

We characterize seasonal site fidelity derived from resighting data the same way as for the satellite-tracking data, i.e. whether birds re-visited the same northward migration sites during the subsequent southward migration. We calculated the proportion of individuals seen at the same Yellow Sea stopping site during both northward and southward migration. We characterize between-year site fidelity during northward migration by calculating the proportion of individuals seen at the same Yellow Sea site in ≥ 2 northward migrations. We compare these proportions between the two species by Fisher’s exact tests. All data analyses were performed in R version 3.6.2 [49]. We used *p* < 0.05 to establish statistical significance.

## Results

**Fig. 1:**
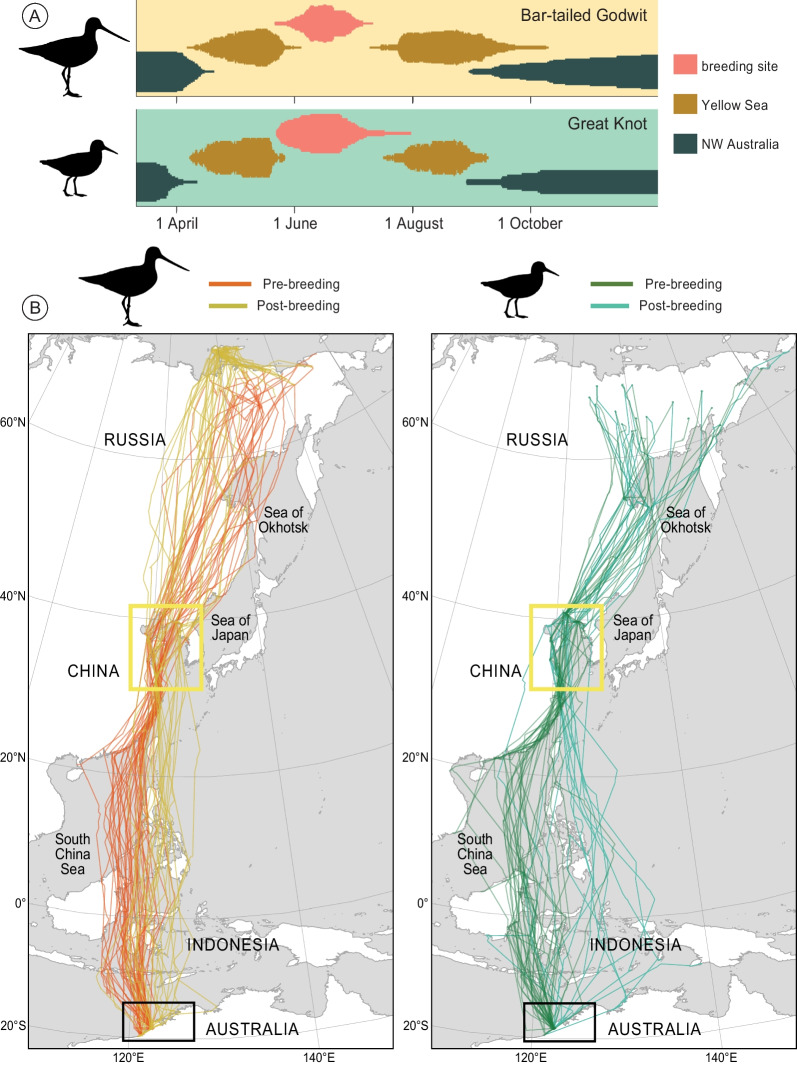
(**A**) Occurrence at non-breeding site (Northwest Australia), staging area (Yellow Sea) and breeding areas for satellite-tagged Bar-tailed Godwits (*Limosa lapponica*) and Great Knots (*Calidris tenuirostris*) in 2015–2017. Height of bars corresponds to the number of individuals. (**B**) Migration tracks of satellite-tagged Bar-tailed Godwits (left) and Great Knots (right) in 2015–2017. Black rectangle indicates Northwest Australia and yellow rectangle indicates the Yellow Sea study area

Within the annual cycle of the Bar-tailed Godwits and Great Knots, both species spent the most time in Northwest Australia, their non-breeding area (Fig. 1A). During their migration from Northwest Australia to breeding areas in the East Russian Arctic and back (Fig. 1B), they spent the longest period along the Yellow Sea coast (Fig. 1A). In both Northwest Australia and the Yellow Sea, the occurrence of the two species strongly overlapped in time (Fig. 1A).

### Site fidelity in the non-breeding period

**Fig. 2:**
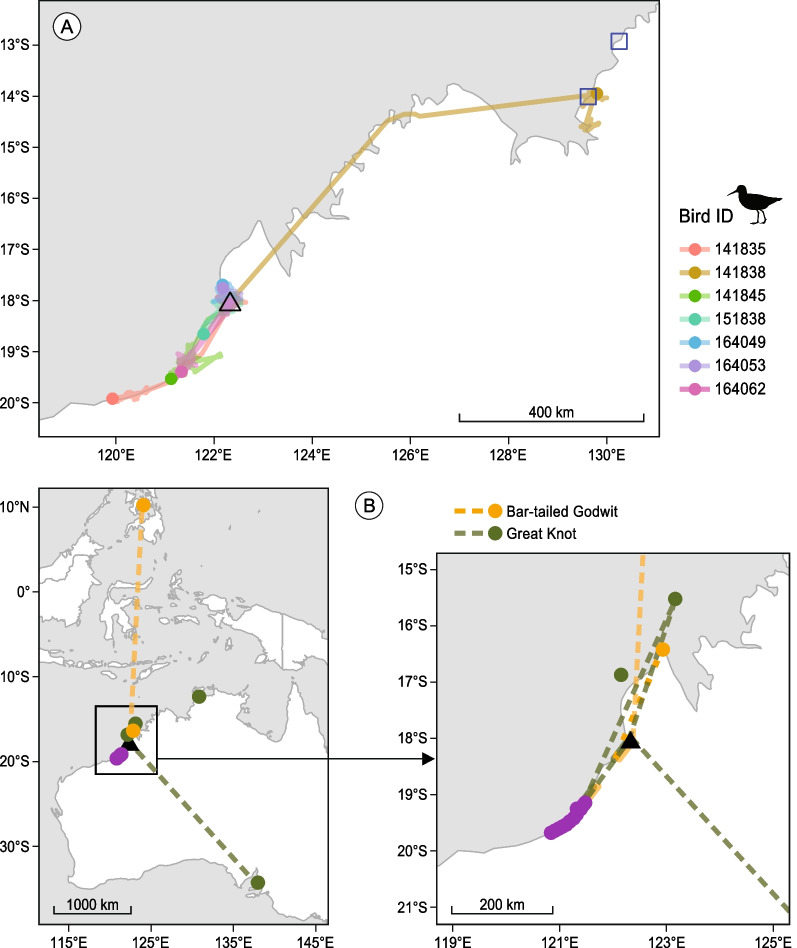
(**A**) Movements of satellite-tracked Great Knots out of Roebuck Bay during the non-breeding period in Northwest Australia, 2014–2017. The triangle denotes Roebuck Bay where individuals were marked. Squares denote non-breeding sites of two individuals that did not return to Roebuck Bay after a complete migration to the breeding grounds. (**B**) Resighting locations of individually marked Great Knots (green) and Bar-tailed Godwits (orange) banded in Roebuck Bay. Triangle denotes Roebuck Bay and purple polygon denotes Eighty Mile Beach. Dotted line connects sites where the same individual was resighted across years. Figure to the right is a zoomed-in version of the area enclosed in the square

In the non-breeding period, none of the 24 satellite-tracked Bar-tailed Godwits moved out of Roebuck Bay, while seven out of 41 (17%) Great Knots moved in and out of Roebuck Bay (Fig. 2A, Table 1). Within-season movement patterns for Great Knots were quite varied. Two birds moved to Willie Creek about 20 km north of Roebuck Bay and one of the two returned briefly to Roebuck Bay. One bird moved south to Bidyadanga (80 km) for less than one day and returned to Roebuck Bay. Of those that moved to Eighty Mile Beach (ca. 170–320 km south), two stayed there and one went back-and-forth twice before finally returning to Roebuck Bay in early March. One bird moved north to Northern Territory, Australia (ca. 920 km north) and stayed there until 23 May when the tag ceased reporting. Four out of these seven Great Knots departed from Roebuck Bay during northward migration, but none were tracked for a complete return migration. Among the 34 Great Knots that we detected only in Roebuck Bay during the non-breeding period, 10 reached the breeding grounds and were tracked until October, in which eight returned to Northwest Australia and two overwintered in the Northern Territory (Table 1, Fig. 2A). Among the 24 Bar-tailed Godwits, nine reached the breeding grounds and all returned to Roebuck Bay (Table 1).

**Table 1:** Site fidelity during the non-breeding period for Bar-tailed Godwits *Limosa lapponica* and Great Knots *Calidris tenuirostris*, as measured from satellite tracking and resighting data

	Bar-tailed Godwit	Great Knot	
*Satellite-tracking data*			
Percentage of individuals that remained at one site during entire non-breeding period	100% (n = 24)	83% (n = 41)	*p* = 0.04*
Percentage of individuals that returned to same non-breeding site after migration	100% (n = 9)	80% (n = 10)	*p* = 0.47
*Resighting data*			
Percentage of individuals resighted at *n* sites within a non-breeding period			
1 site	97.3% (624 birds)	93.8% (727 birds)	
Only Roebuck Bay	617 birds	690 birds	
Only Eighty Mile Beach	7 birds	36 birds	
Only Darwin	0	1 bird	
2 sites (Roebuck Bay and Eighty Mile Beach)	2.7% (17 birds)	6.2% (48 birds)	*p* = 0.001*

Differences between the two species were tested by Fisher’s exact tests

*p < 0.05

The resighting data showed that in both study species, most individuals (> 90%) were resighted at only one site during the non-breeding period. A small percentage were resighted at two sites, namely Roebuck Bay and Eighty Mile Beach, implying that individuals moved between the two sites during the non-breeding period. A higher percentage of Great Knots than Bar-tailed Godwits were resighted within a non-breeding period at the two sites (6.2% vs 2.7%, Fisher’s exact test, *p* = 0.001, Table 1). Four Great Knots and two Bar-tailed Godwits were resighted outside of Roebuck Bay and Eighty Mile Beach during the non-breeding period (Fig. 2B).

### Seasonal site fidelity during migration

**Fig. 3:**
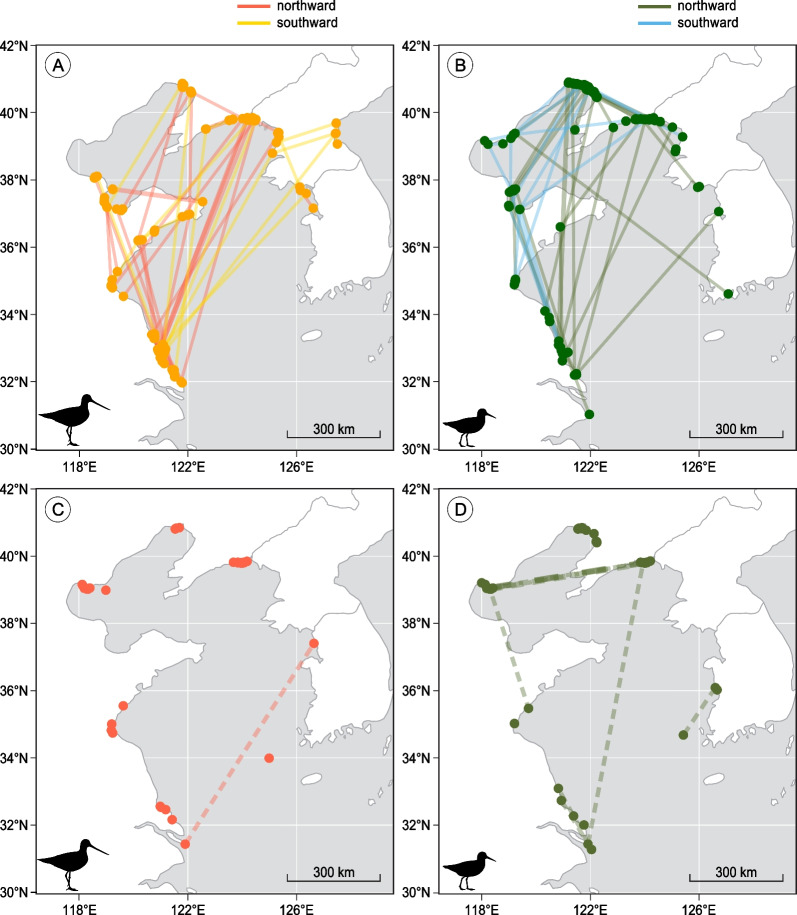
Movements among sites within the Yellow Sea used by satellite-tagged (**A**) Bar-tailed Godwits and (**B**) Great Knots in 2015–2017. Solid lines connect sites visited by an individual within the same northward or southward migration season. Sites within the Yellow Sea where individually marked (**C**) Bar-tailed Godwits and (**D**) Great Knots were resighted during northward migration in 2008–2017. Dashed lines connect sites visited by an individual within the same migration season

**Fig. 4:**
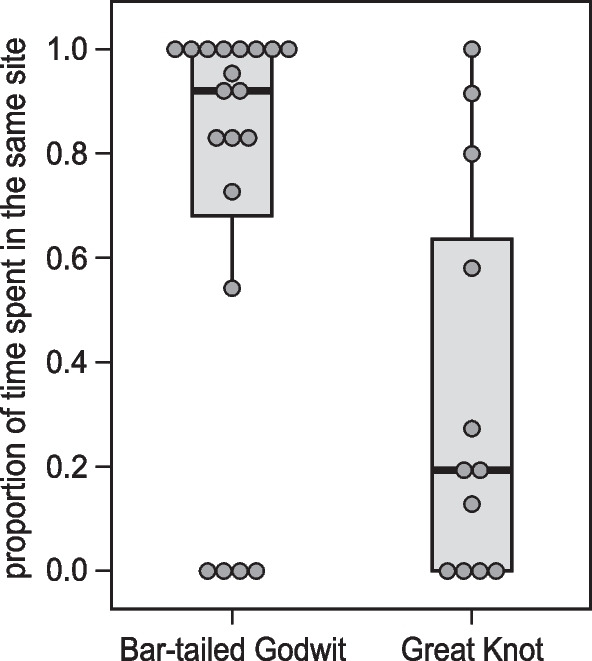
Proportion of time spent at the same sites during northward and southward migration (of total staging duration in southward migration) for satellite-tracked Bar-tailed Godwits (*n* = 20) and Great Knots (*n* = 12) in 2015–2017. Each dot represents the proportion calculated for an individual

Satellite-tracked Bar-tailed Godwits used fewer Yellow Sea sites than Great Knots (median = 2 sites vs. 3 sites) during both northward and southward migration (Table 2, Fig. 3A,B). Sixteen of the 20 (80%) tracked Bar-tailed Godwits, and 8 of the 12 (67%) tracked Great Knots, re-visited the same site(s) during southward migration as used during northward migration. Also, Bar-tailed Godwits stayed proportionally longer at the re-visited sites than Great Knots (92% vs 19%, fractional regression, *p* = 0.01, Table 2, Fig. 4).

**Table 2:** Site fidelity during the migration period in the Yellow Sea for Bar-tailed Godwits *Limosa lapponica* and Great Knots *Calidris tenuirostris*, as measured from satellite tracking data and resighting data

	Bar-tailed Godwit	Great Knot	
*Satellite tracking data*			
Median number of sites used per individual:			
Northward	2 (range: 1–3)	3 (range: 1–4)	
Southward	2 (range: 1–3)	2.5 (range: 1–4)	
Percentage of individuals visiting the same sites during northward and southward migration	80% (n = 20)	67% (n = 12)	*p* = 0.43
Percentage of time spent in the repeatedly visited sites (of total staging duration in southward migration)	Median = 92%	Median = 19%	*p* = 0.01*
*Resighting data*			
Percentage of individuals resighted at *n* Yellow Sea site(s) within a northward migration			
1 site	99.2% (132 birds)	97.7% (333 birds)	*p* = 0.45
2 sites	0.8% (1 bird)	2.1% (7 birds)	
3 sites	0	0.3% (1 bird)	
Percentage of individuals resighted at the same Yellow Sea site during both northward and southward migration	90.9% (n = 11)	63.6% (n = 11)	*p* = 0.31
Percentage of individuals resighted at the same Yellow Sea site in > = 2 northward migrations	98.0% (n = 102)	89.5% (n = 267)	*p* = 0.005*

Differences between the two species in proportions of individuals were tested by Fisher’s exact tests. The difference in percentage of time spent in the repeatedly visited sites was tested by fractional regression

*p < 0.05

From the resighting data, within the northward migration season most individuals (> = 98% for both species) were observed at only one of the Yellow Sea sites (Table 2, Fig. 3C,D). The percentage of individuals seen at two or more sites was not significantly different between Bar-tailed Godwits and Great Knots within a season (0.8% vs. 2.4%, Fisher’s exact test, *p* = 0.45). Pulling data from all the years together, among those individuals resighted during both northward and southward migration, 10 out of 11 Bar-tailed Godwits and 7 out of 11 Great Knots were seen at the same northward stopping site during southward migration of the same year. Across years, more Bar-tailed Godwit individuals were seen at the same northward migration Yellow Sea stopping sites (98.0%) than Great Knots (89.5%; Fisher’s exact test, *p* = 0.005).

## Discussion

Our findings based on both satellite tracking and resighting data revealed high site fidelity in both study species, with the majority of individuals using only one site during the non-breeding season and returning to the same stopping sites during north- and southward migration. However, the pattern of Bar-tailed Godwits being more site-faithful than Great Knots holds across places within the non-breeding season. In the non-breeding period, both data types showed that Bar-tailed Godwits are significantly more site faithful than Great Knots and less likely to move between sites. During migration, seasonal site fidelity (the proportion of individuals visiting the same sites during northward and southward migration) did not differ significantly between the two species based on both data types, although in absolute terms the proportion of site-faithful Bar-tailed Godwits was higher than that of Great Knots. However, the degree of site fidelity, measured by the proportion of time spent at the repeatedly visited sites, was significantly higher for Bar-tailed Godwits than Great Knots. This pattern also holds for the fidelity across northward migrations measured based on resighting data. In two published studies comparing site fidelity between shorebird species, one conducted across the entire country of New Zealand [50] and one at Moray Basin, Scotland [51], both concluded that the Bar-tailed Godwits were more site-faithful than Red Knots (*Calidris canutus,* a sister species of the Great Knot). These studies and our current study together suggest that the difference in the degree of site fidelity between Knots and Godwits is consistent across places. As these two species co-occur in the same habitat, the difference in site fidelity between the Knot and Godwit might reflect different spatial and year-to-year predictability of their preferred prey [52, 53]. While the spatial and temporal variation in prey of the Knot is better studied (e.g. [54]), little is known about how prey of Bar-tailed Godwits varies in space and time. Long-term measurements of prey distributions (e.g. [55, 56]) would allow testing hypotheses regarding the relationship between site fidelity and prey variability. Our data do not allow an investigation of whether site fidelity is an individual-specific trait (i.e. if certain individuals are consistently more faithful both in the Yellow Sea and at Northwest Australia). This is because there was no between-individual variation in site fidelity among Bar-tailed Godwits (all birds were faithful to Northwest Australia). And, for Great Knots, the individuals that were not site faithful in Northwest Australia were not tracked past the Yellow Sea during southward migration (resulting in no data on seasonal site fidelity in the Yellow Sea for those individuals).

While the mark-resighting data and satellite tracking data both showed that Bar-tailed Godwits were the more site-faithful species, mark-resighting data alone underestimated the proportion of individuals of both species that moved between sites during the non-breeding season (Table 1) and the number of sites birds used in the Yellow Sea (Table 2). For example, during the northward migration, satellite tracking data showed individual Great Knots used three sites in the Yellow Sea, and Bar-tailed Godwit used two, while the mark-resighting data indicated that most individuals only used one site. This pattern is likely an outcome that, constrained by logistics, many sites visited by the birds were unsurveyed or only sporadically surveyed (as illustrated for Great Knots in [23]); and at the surveyed sites, ground observers could have missed some flocks or some marked individuals within dense flocks. Although tracking individual birds with satellite transmitters does have its limitations, e.g. the handicap of carrying a tag can alter migration patterns in some cases [57], tags do provide a more representative measure of site use and fidelity than mark-resighting data. However, since mark-resighting data is still the most prevalent data set for most shorebird species in the East Asian-Australasian Flyway and elsewhere, it can be utilized for multi-species comparisons of site fidelity, bearing in mind that the results should be interpreted as a relative measure of site fidelity.

### Site fidelity and the response to habitat loss and deterioration in the Yellow Sea

During our study, shorebird habitats in Northwest Australia remained stable, whereas habitats in the Yellow Sea underwent significant loss and deterioration [25–28, 58]. Notably, a major event of habitat deterioration that occurred just prior to our study may have impacted Great Knots more heavily than Bar-tailed Godwits. In April 2006, ~ 290 km^2^ of tidal flats were impounded by the closure of the 33 km-seawall at Saemangeum (35.8°N, 126.6°E) in South Korea. This area supported 20–30% of the world population of Great Knots during both northward and southward migration in the late 1990s to early 2000s [59]. About 100,000 Great Knots disappeared from Saemangeum and the adjacent Geum Estuary and no substantial increase in Great Knot numbers was observed at other nearby staging sites [60]. If these missing birds mostly died, Great Knots should show a particularly severe decline in survival and population size. However, the subsequent rates of decline in adult survival (2006–2012) and in population size (1993–2012) did not differ between Knots and the less-habitat affected Godwits [28, 29]. One possible explanation for this pattern might be that the lower site faithfulness of Great Knots allowed them to respond to this dramatic loss of habitat by moving to alternative sites, which might have helped to soften the impact.

S﻿imilar processes could have happened when habitat deteriorated at other main staging sites for Bar-tailed Godwits and Great Knots. One well-documented habitat deterioration event took place at Yalu Jiang Estuary of the Yellow Sea [40]. Yearly monitoring of the macrobenthic community in 2011–2016 showed that the population of *Potamocorbula laevis*, a main bivalve prey of shorebirds*,* had drastically declined starting in 2013; the very high density in 2011 (708 ind/m^2^) had declined by > 99% in 2016 [54]. This drastic change likely profoundly impacted the Great Knot, a mollusk specialist, and less so the Bar-tailed Godwit which also feeds on polychaetes [61]. The lower site fidelity of Great Knots compared to Bar-tailed Godwits in the Yellow Sea might partly reflect that the Great Knots were more affected by habitat deterioration and thus, needed to move to alternative sites more often. However, the same between-species difference in site fidelity was also found in Northwest Australia. This leads to the question of whether the individual Great Knots that survived habitat deterioration events are the individuals with the tendency for lower site fidelity. If so, this could contribute to the site fidelity patterns that we measured at the population level. And, if site fidelity has a heritable component, events selecting for low site fidelity individuals would lead to a decrease in site fidelity level over generations. Of particular interest would be the situation of habitat gains rather than losses: if some Yellow Sea habitats are being restored in the future, would less site faithful individuals be faster at discovering restored sites? Thus, would populations with a higher proportion of low-site fidelity individuals recover more rapidly?

## Conclusions

To answer the questions raised above, site fidelity exhibited by different species should be measured over periods of positive or negative changes in habitat quality. Our study provides essential insights towards such a comparative approach. We show that differences in site fidelity between species are consistent across satellite tracking and resighting data. While satellite tracking data provides more fine-scaled patterns [62], these data are more costly to collect and have not been implemented in many species. Therefore, long-term resighting data are a viable alternative for quantifying site fidelity. As differences in site fidelity between the Knots and Godwits persisted in both the non-breeding and migration periods, further studies with multiple species would verify if a migratory species’ site fidelity in one location can provide insights into its site fidelity in the whole annual cycle. Ultimately, concurrent measurements of demographic rates (survival and recruitment) and population trends would be needed to understand the significance of site fidelity variation in population response to human-induced environmental changes.

### Supplementary Information

**Additional file 1:** Surveyed sites along the Chinese coast in April and May 2015–2017.

## Data Availability

The datasets analysed during the current study are available from the corresponding author on reasonable request.
