# Supplementary material for: Site fidelity of migratory shorebirds facing habitat deterioration: insights from satellite tracking and mark-resighting
Source: Mov Ecol. 2023 Dec 21;11:79. doi: 10.1186/s40462-023-00443-9 (PMC10740345; doi:10.1186/s40462-023-00443-9)
Supplement: Supplementary file 1 — Additional file 1: Surveyed sites along the Chinese coast in April and May 2015–2017. [file 40462_2023_443_MOESM1_ESM.pdf]

Additional File 1

Article title: Site fidelity of migratory shorebirds facing habitat deterioration: insights from satellite tracking and mark-resighting

Ying-Chi Chan, David Tsz Chung Chan, T Lee Tibbitts, Chris J Hassell, Theunis Piersma

Table A1.1. Surveyed sites along the Chinese coast in April and May 2015–2017.

**Site name and Province**	**Latitude (°N)**	**Longitude (°E)**	**Survey conducted**		
			2015	2016	2017
Panjin, Liaoning Province	40.76	121.86		x	x
Gaizhou, Liaoning Province	40.45	122.23	x	x	x
Diaokou, Shandong Province	38.09	118.58		x	x
Changyi, Shandong Province	37.14	119.49		x	x
Lianyungang, Jiangsu Province	35.01	119.21	x	x	x
Xinchuangang, Jiangsu Province	32.63	120.99		x	x
Tongzhou, Jiangsu Province	32.18	121.43	x	x	x
Qidong, Jiangsu Province	32.00	121.78		x	x
Cixi, Zhejiang Province	30.40	121.19			x
Linhai, Zhejiang Province	28.73	121.67			x
Ruian, Zhejiang Province	27.73	120.76			x
Raoping, Guangdong Province	23.59	117.14		x	x
Hailingdao, Guangdong Province	21.71	111.94			x
Dongliaodao, Guangdong Province	20.83	110.38		x	x
